# Nestin expression involves invasiveness of esophageal carcinoma and its downregulation enhances paclitaxel sensitivity to esophageal carcinoma cell apoptosis

**DOI:** 10.18632/oncotarget.17774

**Published:** 2017-05-10

**Authors:** Jinghang Zhang, Jiateng Zhong, Jian Yu, Jinsong Li, Wenyu Di, Ping Lu, Xiaoyu Yang, Weixing Zhao, Xianwei Wang, Wei Su

**Affiliations:** ^1^ Department of Pathology, The First Affiliated Hospital of Xinxiang Medical University, Xinxiang 453003, P.R. China; ^2^ Henan Key Laboratory of Medical Tissue Regeneration, Xinxiang Medical University, Xinxiang 453003, P.R. China

**Keywords:** nestin, paclitaxel, apoptosis, metastasis, esophageal carcinoma

## Abstract

Paclitaxel has been generally used to treat primary and metastatic esophageal carcinoma. It has been shown that nestin is highly expressed in esophageal carcinoma and that there is a strong association of nestin expression with poor prognosis in esophageal carcinoma patients. In this study, using immunohistochemistry, *in situ* hybridization and Western blotting we demonstrated that nestin was overexpressed in the invasive esophageal carcinoma. To further elucidate whether nestin inhibition could enhance paclitaxel sensitivity to esophageal carcinoma cells, we applied nestin siRNA in esophageal squamous cell carcinoma Eca-109 cells. Flow cytometry and TUNEL staining both showed that combination of paclitaxel treatment and nestin knockdown resulted in greater induction of apoptosis of esophageal carcinoma cells as compared with the cells transfected with control siRNA (also treated with paclitaxel). This study indicates that nestin knockdown enhances chemotherapeutic sensitivity of paclitaxel to esophageal carcinoma, and suggests that silencing of nestin could be a valuble therapeutic approach for enhancing drug sensitivity and thereby improving the treatment outcome of esophageal carcinoma patients.

## INTRODUCTION

Esophageal carcinoma is the eighth most frequent malignant tumors worldwide [1]. Currently, surgery is the primary treatment, but the 5-year survival of patients with metastatic esophageal carcinoma managed with surgery remains poor, less than 20–40% [2]. Metastasis is a complex process that involves activation of extracellular matrix proteases, cell motility, intravasation to vessels, travel via the circulatory system, and survival and establishment of secondary tumors in new microenvironment [3, 4].

Paclitaxel, a microtubule-stabilizing drug, has been widely used as a single agent or in combination therapy with other chemotherapeutic agents to treat primary and metastatic esophageal carcinoma [5]. However, esophageal carcinoma is generally insensitive to anticancer drugs, which makes chemotherapeutic treatment difficult in the clinic. Therefore, discovery of the mechanisms underlying drug resistance is of great importance in the development of new strategies for treatment of drug-resistant tumors. The development of drug-resistance is ascribed to the expression of drug resistance related genes in tumor cells. A previous study has revealed that various cellular pathways may participate simultaneously in drug-resistance. Increased repair of drug-induced DNA damage, blocked apoptosis, disruptions in the signal pathways, and alterations of factors involved in cell cycle contribute to the development of drug-resistance. Nestin is a cytoskeleton-associated class VI intermediate filament protein that was originally identified in stem cells and progenitor cells in the central nervous system, as well as in peripheral organs [6–8]. It has been shown to contribute to drug resistance in P-glycoprotein-dependent manner [9].

Recently, nestin has also been detected in esophageal carcinoma [10]. In this study, we demonstrated that the expression level of nestin was correlated the invasiveness of esophageal carcinoma and inhibition of nestin enhanced paclitaxel sensitivity to apoptosis of esophageal carcinoma cells.

## RESULTS

### Overexpression of nestin in esophageal carcinoma, which is related to tumor invasion

**Figure 1:**
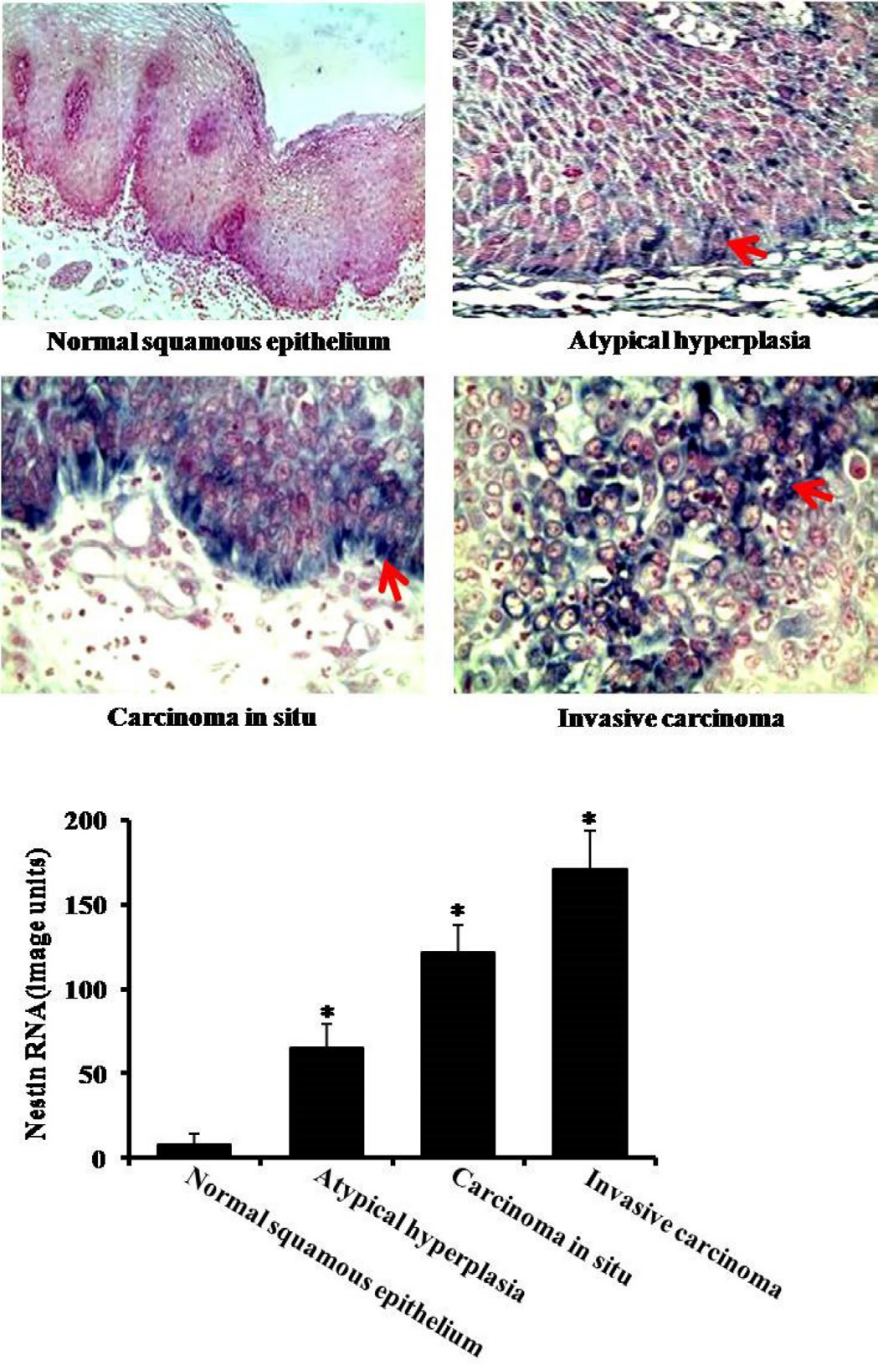
Expression of nestin in esophageal carcinoma by *in situ* hybridization There are four groups: normal suquamous epithelium tissues, atypical hyperlasia, esophageal carcinoma *in situ* and invasive esophageal carcinoma. Arrows indicate nestin expression. *P < 0.05 vs. normal squamous epitheliu group.

To examine the expression of nestin in esophageal carcinoma, we used *in situ* hybridization to detect nestin mRNA level. Compared to normal squamous epithelium tissues, the expression of nestin increased in squamous epithelium atypical hyperplasia, esophageal carcinoma *in situ* and invasive esophageal carcinoma (Figure 1). The highest expression of nestin was observed in invasive esophageal carcinoma (Figure 1).

### The protein expression of nestin in esophageal carcinoma

**Figure 2:**
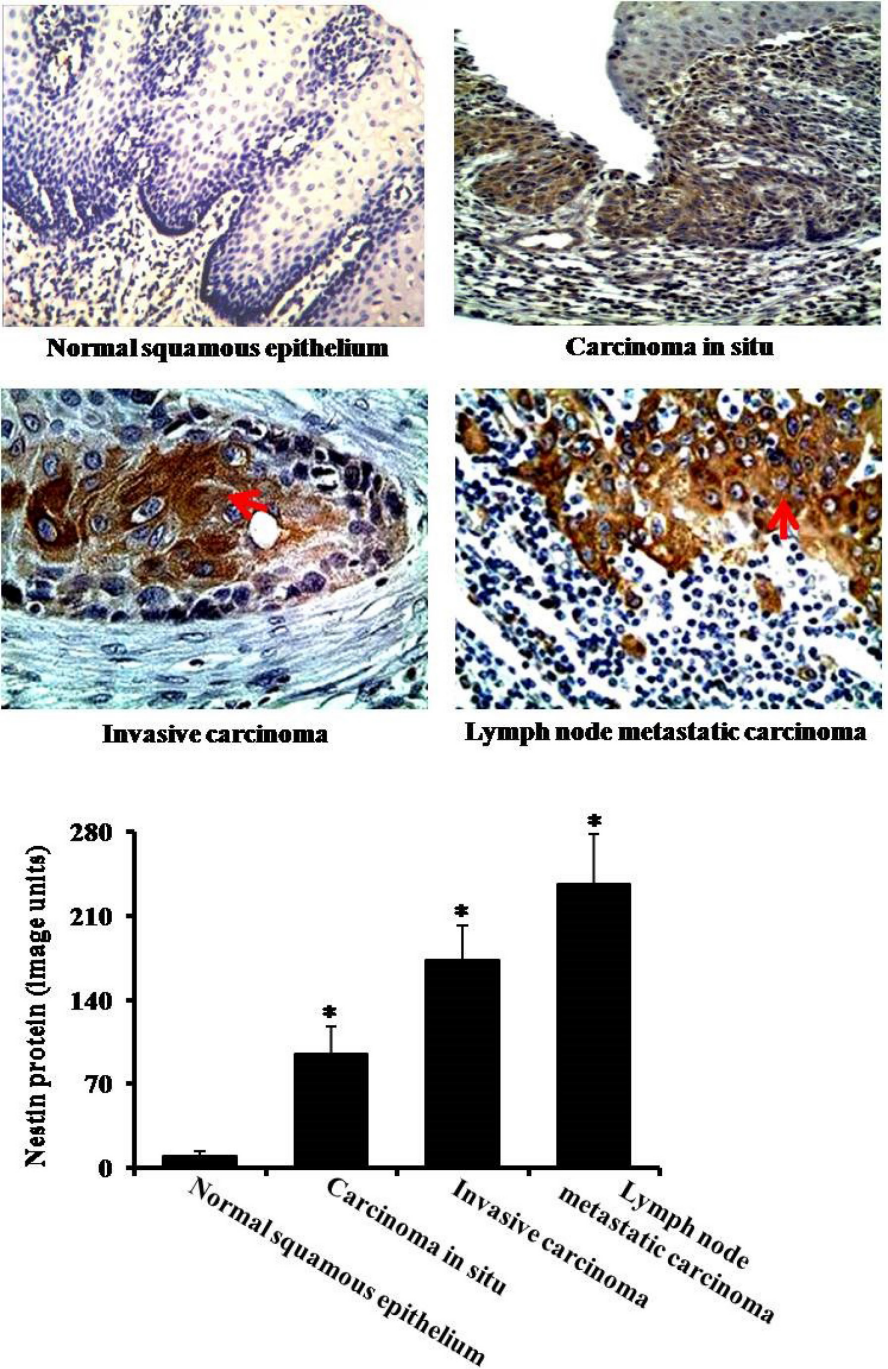
Immunohistochemical analysis of nestin There are four groups: nomal squamous epithelium, carcinoma *in situ*, invasive carcinoma and lymph node metastatic carcinama. Arrows indicate nestin expression. *P < 0.05 vs. normal squamous epithelium group.

**Figure 3:**
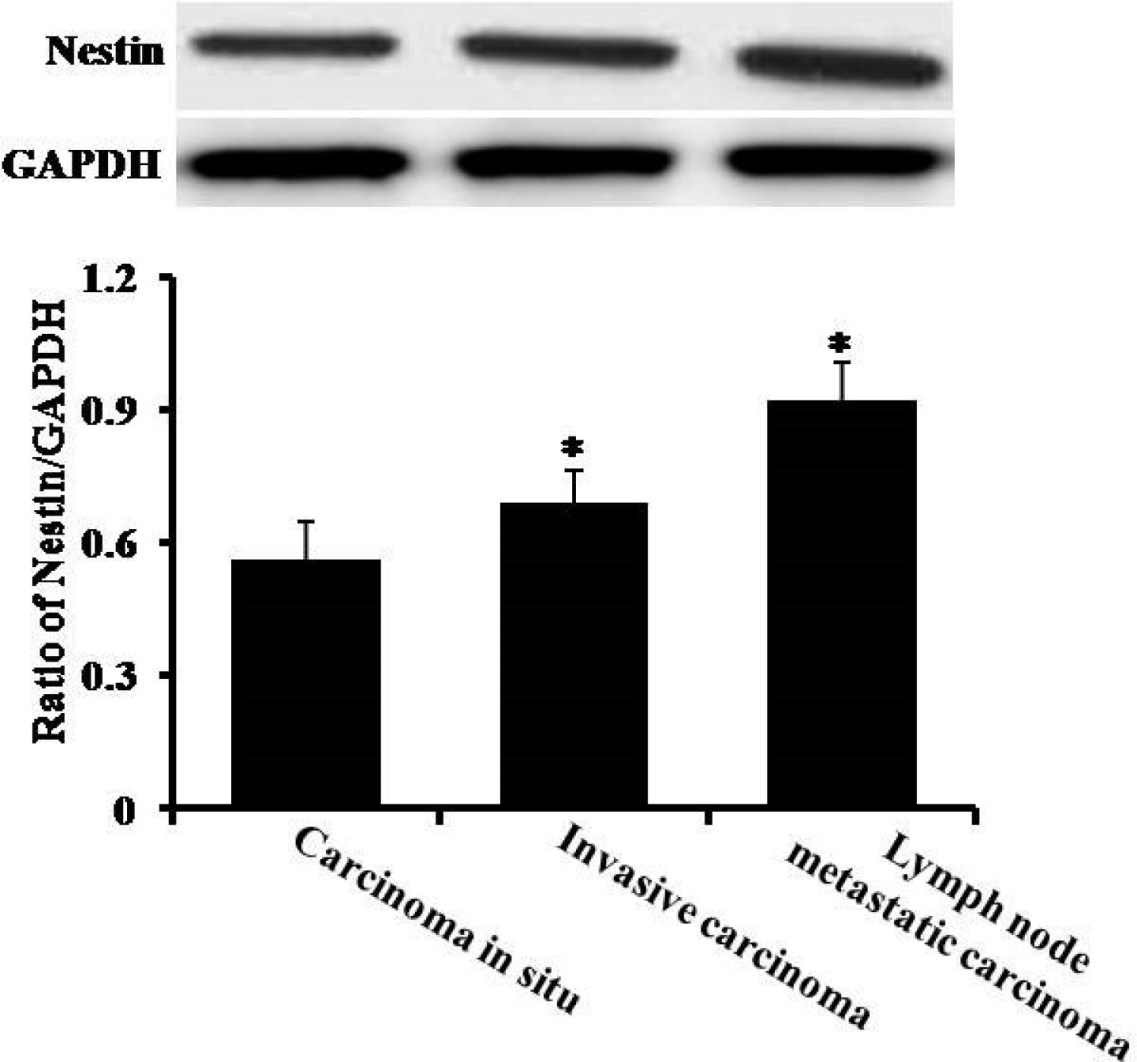
The analysis of protein content by Western blotting There are three groups: carcinoma *in situ*, invasive carcinoma and lymph node metastatic carcinoma. *P < 0.05 vs. carcinoma *in situ* group.

Next, we analyzed protein expression of nestin in nomal squamous epithelium, carcinoma *in situ*, ivasive carcinoma and lymph node metastatic carcinama. The immunohistochemistry data showed that the expression levels of nestin were much higher in esophageal carcinoma *in situ*, esophageal invasive carcinoma and lymph node metastatic carcinoma as compared to normal squamous epithelium group (Figure 2). In line with immunohistochemistry results, the Western blotting data also showed significant increases of nestin in esophageal carcinoma *in situ*, esophageal invasive carcinoma and lymph node metastatic carcinoma (Figure 3). The highest expression of nestin was in the invasive carcinoma and lymph node metastatic carcinama (Figures 2 and 3).

### The expression of nestin in non-metastasized and metastasized esophageal carcinoma

**Figure 4:**
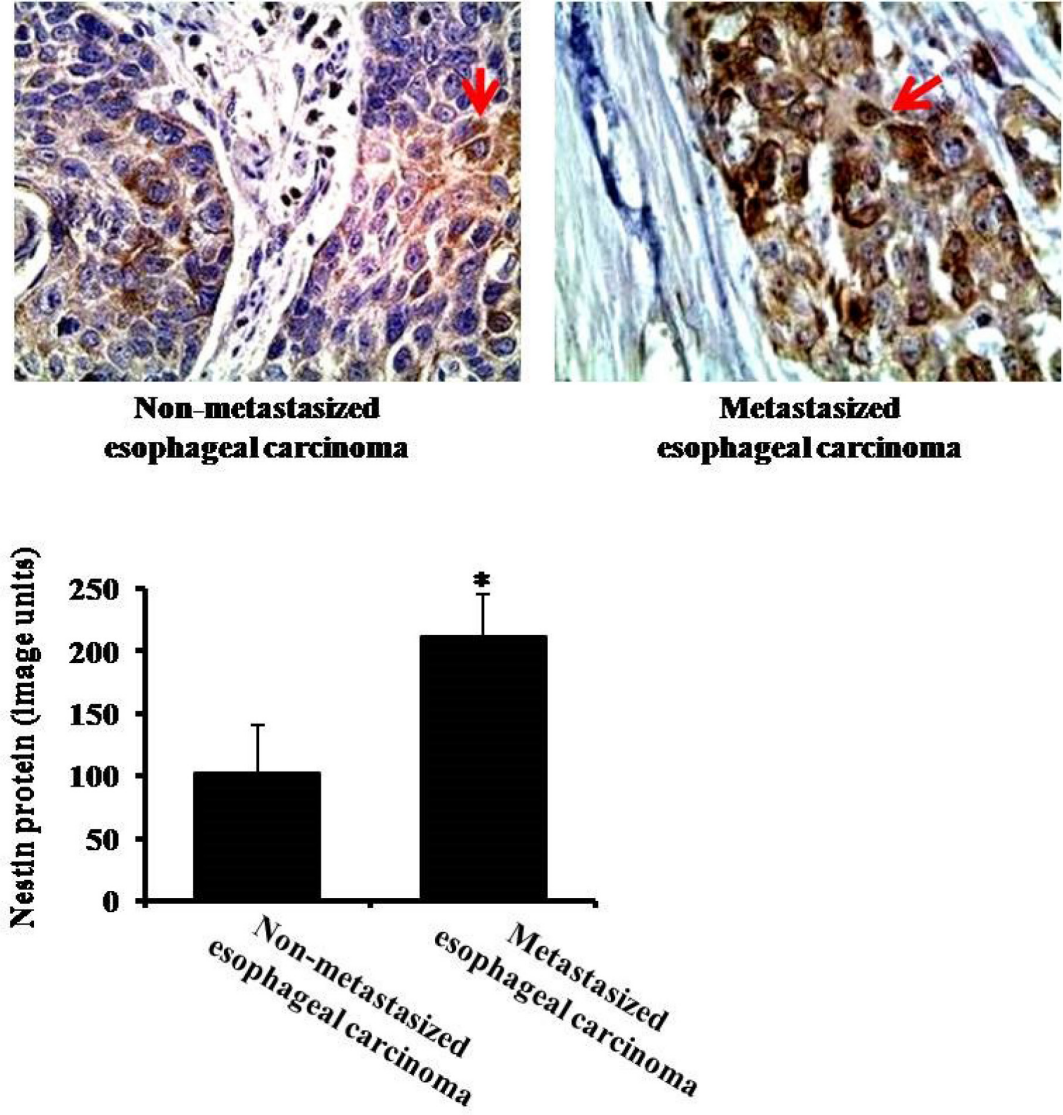
The expression of nestin in non-metastasized (without lymph node metastasis) and metastasized (with lymph node metastasis) esophageal carcinoma by immunohistochemistry Arrows indicate nestin expression. *P < 0.05 vs. Non-metastasized esophageal carcinoma group.

To explore the different expression of nestin in non-metastasized esophageal carcinoma and metastasized esophageal carcinoma, we did nestin immunohistochemistry staining. Compared to non-metastasized esophageal carcinoma (without lymph node metastasis), the expression of nestin was markedly higher in metastasized esophageal carcinoma (with lymph node metastasis) (Figure 4).

### Inhibition of nestin enhances paclitaxel sensitivity of esophageal carcinoma cells

**Figure 5:**
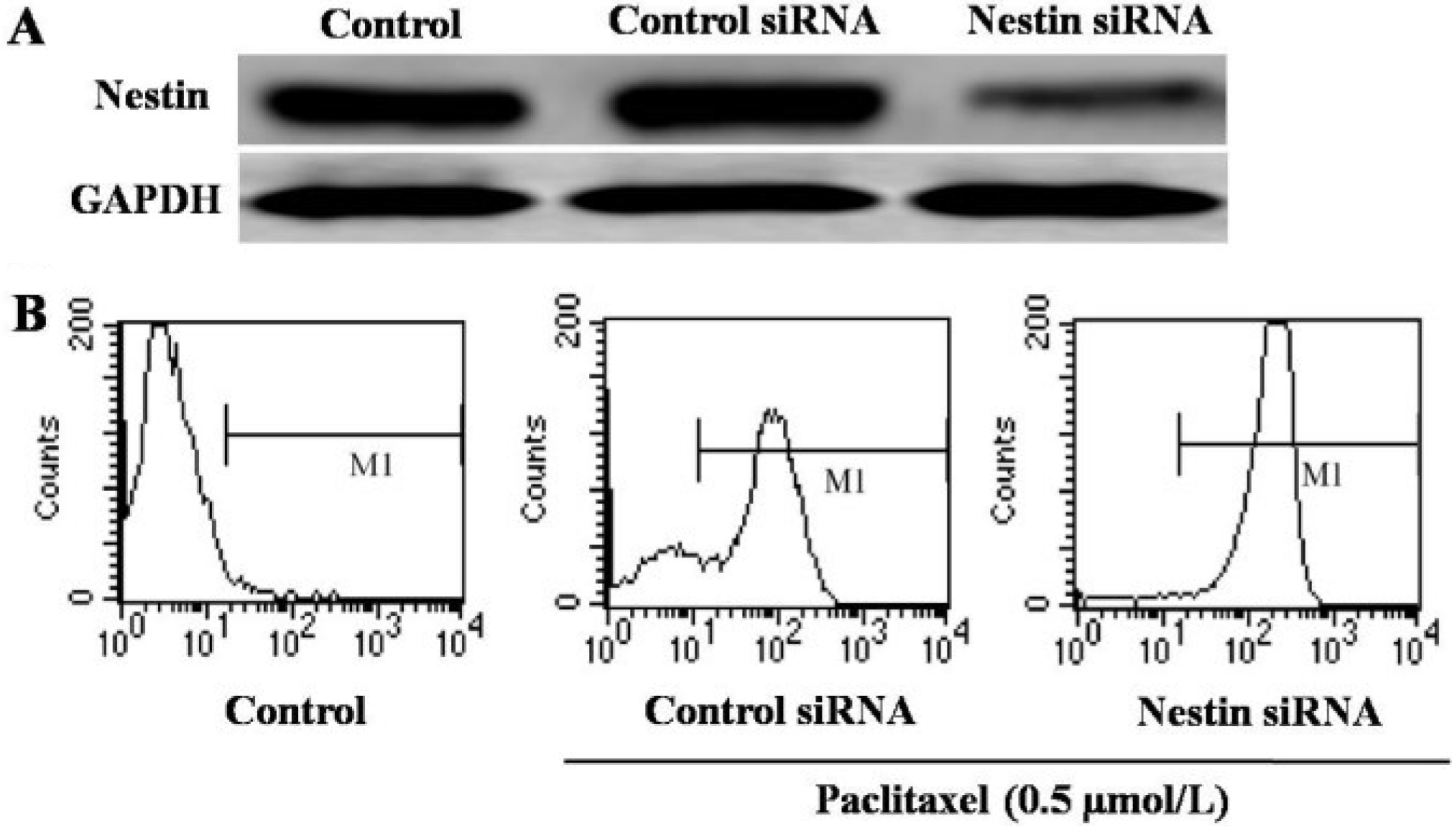
Analysis of apoptosis of Eca-109 cells (**A**) Western blotting analysis of the expression of nestin in Eca-109 cells treated with control siRNA or nestin siRNA. (**B**) Detecton of apoptosis by flow cytometry. There are three groups: untreated control, control siRNA and nestin siRNA. As detecton of apoptosis, the cells in control siRNA and nestin siRNA groups were treated with 0.5 µmol/L paclitaxel.

To investigate the effect of nestin on apoptosis in esophageal carcinoma cells, we used nestin siRNA to inhibit nestin expression in Eca-109 cells. As shown in Figure 5A, nestin siRNA application significantly reduced the expression of nestin. Next, we analyzed the apoptotic response to paclitaxel (0.5 μmol/L) in cells transfected with either control or nestin siRNA. Compared to cells transfected with control siRNA, nestin siRNA significantly increased apoptosis (Annexin V positive) of Eca-109 cells (Figure 5B).

**Figure 6:**
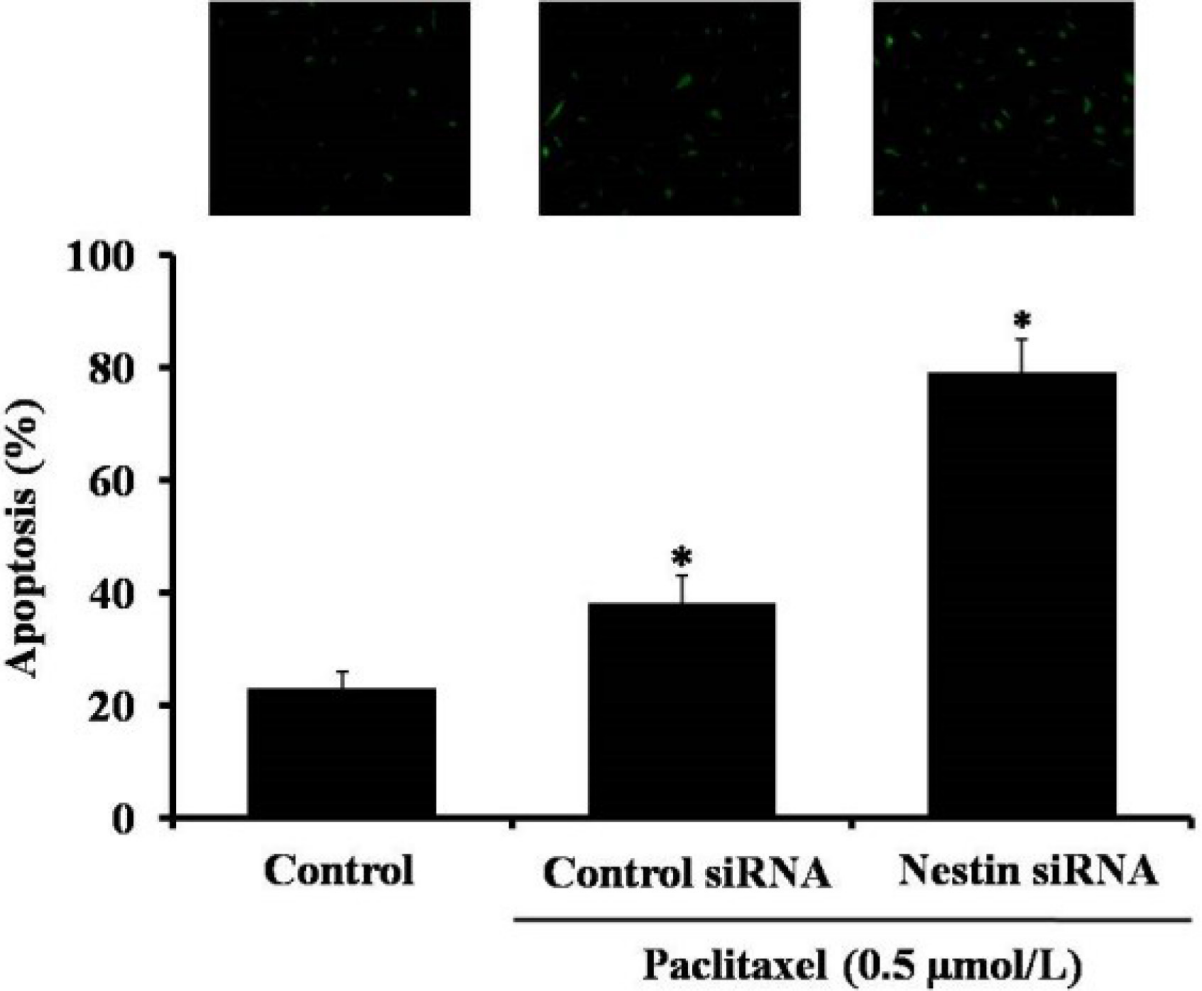
Detection of apoptosis in paclitaxel treated Eca-109 cells by TUNEL assay There are three groups: untreated control, control siRNA and nestin siRNA. The cells in control siRNA and nestin siRNA grooups were treated with 0.5 µmol/L paclitaxel. Data are presented as mean ± SD, *n* = 3, *p < 0.05 versus control group.

To further confirm our result, we also used TUNEL assay. In agreemment with flow cytometry data, the inhibition of nestin by siRNA increased the number of apoptotic cells induced by paclitaxel treatment (Figure 6).

## DISCUSSION

Paclitaxel has been used clinically to treat many carcinomas including esophageal carcinoma by promoting microtubule polymerization [12]. However, in many cases esophageal carcinoma relapsed due to development of resistance rendering paclitaxel ineffective. Studies have also shown that numerous genes are involved in paclitaxel drug-resistance [13]. Recent reports showed that high expression of nestin correlated with malignant characteristics in several carcinomas and suggested that elevated content of nestin in cancer cells was linked to greater aggressiveness and poor prognosis [14–17]. However, there has been little research on the relations between nestin expression and the therapeutic efficacy of paclitaxel in the esophageal carcinoma in the past years.

Here, we demonstrate that the expression of nestin is significantly higher in invasive esophageal carcinoma than in carcinoma *in situ*. This result suggests that nestin may promote the progress and metastasis of esophageal carcinoma and is, therefore, an atrractive potential target for cancer therapy.

Furthermore, we were interested whether proconditioning the cells through nestin silencing could augment the chemotherapeutic sensitivity to paclitaxel. As a highly effective chemotherapy agent, paclitaxel has been extensively used to treat patients with paclitaxel [17]. Consequently, paclitaxel suppresses tumor growth but sometimes its efficacy is compromised by the development of resistance. We tested the effects of paclitaxel on apoptosis of Eca-109 cells transfected with nestin siRNA using flow cytometry and TUNEL assay. Our results demonstrated that the combination of paclitaxel with nestin siRNA could improve apoptotic responses of Eca-109 cells. These results suggest that nestin contributes to the evasion of apoptosis in Eca-109 cells.

In summary, this study indicates that knockdown of nestin enhances the chemotherapeutic sensitivity to paclitaxel. Our study offers convincing evidence that treatment with paclitaxel combined with nestin inhibition may improve chemosensitivity of patients with esophageal carcinoma.

## MATERIALS AND METHODS

### Tissue specimens and materials

Esophageal carcinoma specimens were collected from patients diagnosed at the Department of Pathology of the First Affiliated Hospital of Xinxiang Medical University. The study was approved by the Ethical Committee of Xinxiang Medical University, and conformed to the Declaration of Helsinki and the Good Clinical Practice Guidelines. All patients participating in this project provided written informed consent.

Dulbecco’s modified eagle media (DMEM) and Fetal bovine serum (FBS) were purchased from GIBCO. The anti-nestin antibody was purchased from Millipore and anti-GAPDH antibody was purchased from Santa Cruz Biotechnology.

### Cell culture

Eca-109 cells were obtained from the Cell Bank of the Chinese Academy of Sciences and cultured in DMEM with 10% FBS under standard culture conditions (37°C and 5% CO_2_).

### *In situ* hybridization (ISH)

The analysis of nestin gene change was performed with a ISH probe kit (Abbott Molecular Inc.). Slides were treated with xylene and incubated with proteinases K at 56°C over night to expose RNA. Next, slides were dehydrated in ethanol series (70, 85, and 100%, 5 min each step). Denaturation was performed by incubation of the slides in 70% formamide at 80°C for 2 min. After that, hybridization with the RNA probe was performed in humidified chamber overnight. Images were captured digitally with an Olympus BX53 epi-fluorescence microscope.

### Immunohistochemistry

The analysis was performed to detect the protein of nestin in esophageal squamous cell carcinoma (ESCC) tissues by using the Histostain-SP Kit (Zymed-Invitrogen, Carlsbad, CA, USA). Slides were digested with hyaluronidase (5 mg/mL, Sigma, MO, USA) for 15 min at 37°C. Then, the slides were incubated with primary antibody against nestin (1:300 dilution) at 4°C over night. Then, samples were treated with secondary antibody and streptavidin-peroxidase conjugate (Zymed-Invitrogen), followed by standardized development in DAB (3′3-diaminobenzidine). Images were captured by using a Nikon E800 microscope.

### Western blotting

Proteins were extracted from esophageal carcinoma sample or Eca-109 cells and protein concentrations were detected by using the BCA Protein assay. Then, 35 μg protein were separated by 12% SDS-polyacrylamide gel for 90 min and transferred onto PVDF membranes. After that the PVDF membranes were blocked with skim milk (5%) for 120 min at room temperature and incubated with the primary antibodies (nestin or GAPDH) overnight at 4°C. On the second day, PVDF membranes were incubated with secondary antibodies for 30 min. The semi-quantitation of proteins was performed with a Tanon GIS gel imager System.

### siRNA transfection

The specific siRNA nestin sequences: sense (5′GUGCCAGCCUUUCUUAAGATT-3′) and antisense (5′UCUUAAGAAAGGCUGGCACTT-3′); negative control: sensce (5′GCGACGAUCUGCCUAAGAUdTdT-3′) and antisense (5′AUCUUAGGCAGAUCGUCGCdTdT-3′). The siRNA sequences were synthesized by Shanghai Bioengineering Technology Service Company of China. Before transfection, 1 × 10^5^ cells were cultured on 6-well plates in normal medium and were transfected with siRNA using Lipofectamine (Invitrogen, Carlsbad, CA, USA). Cells were harvested for Western blotting after 24 hours post-transfection.

### Flow cytometry for analysis of apoptosis

After transfection for 24 hours, Eca-109 cells were treated with 0.5 µmol paclitaxel for additional 48 hours. Following the degestion and washing with PBS, the cells were incubated at 37°C for 20 min with Propidium iodide (PI, 1 mg/ml, Invitrogen) and Annexin V-FITC (1 mg/ml, Invitrogen). Then, the samples were analyzed by a FACScan flow cytometer (Becton Dickinson, Franklin Lakes, NJ, USA).

### TUNEL assays

TUNEL assay was performed using an *In Situ* Cell Death Detection kit according to the manufacturer’s instructions. Following transfection and treatments with paclitaxel, Eca-109 cells were fixed in 4% (w/v) paraformaldehyde for 20 min and then washed with cold PBS. Cells were incubated with TUNEL reaction mix for 45 min. Apoptotic cells (staining green) were counted in six random fields.

### Statistical analysis

The data were analyzed by one-way ANOVA with Tukey’s post-hoc adjustment for multiple comparisons and Student’s *t* test for two group comparison. *P* < 0.05 was considered to a statistically difference.
